# Supplementary material for: Development of a computer-aided design software for the quantitative evaluation of aesthetic damage
Source: PLoS One. 2019 Dec 18;14(12):e0226322. doi: 10.1371/journal.pone.0226322 (PMC6919621; doi:10.1371/journal.pone.0226322)
Supplement: S1 Code site — 1. (ZIP) [file pone.0226322.s001.zip › [EESC_jr][Projeto]Relatorio_07_21/reports/templates_zip/gentelella-master/vendors/Flot/examples/navigate/index.html]

Flot Examples: Navigation


## Navigation

With the navigate plugin it is easy to add panning and zooming. Drag to pan, double click to zoom (or use the mouse scrollwheel).

The plugin fires events (useful for synchronizing several plots) and adds a couple of public methods so you can easily build a little user interface around it, like the little buttons at the top right in the plot.

Copyright © 2007 - 2014 IOLA and Ole Laursen
